# N6-methyladenosine modification of circCUX1 confers radioresistance of hypopharyngeal squamous cell carcinoma through caspase1 pathway

**DOI:** 10.1038/s41419-021-03558-2

**Published:** 2021-03-19

**Authors:** Ping Wu, Xing Fang, Yalan Liu, Yaoyun Tang, Wei Wang, Xin Li, Yuhua Fan

**Affiliations:** Department of Otorhinolaryngology Head & Neck Surgery; Province Key Laboratory of Otolaryngology Critical Diseases, Xiangya Hospital, Central South University, Changsha, 410008 China

**Keywords:** Radiotherapy, Head and neck cancer

## Abstract

Hypopharyngeal squamous cell carcinoma (HPSCC) is one of the most common malignant tumors in otolaryngology head and neck surgery and is one of the worst prognostic malignant tumors. Endogenous circular RNA (circRNA) is more stable than mRNA, microRNA (miRNA), and long non-coding RNA (LncRNA) in exosomes, plasma, and urine, and participates in gene expression regulation to perform different functions. Therefore, circRNA is expected to become a biomarker and therapy target for many tumors. However, the expression and function of circRNA regulated by N6-methyladenosine (m6A) are still unclear in HNSCC. In this study, we demonstrated that a specific circRNA, circCUX1, was upregulated in HPSCC patients who are resistant to radiotherapy and predicts poor survival outcome. We further found that methyltransferase like 3 (METTL3) mediated the m6A methylation of circCUX1 and stabilizes its expression. Knockdown circCUX1 promotes the sensitivity of hypopharyngeal cancer cells to radiotherapy. In addition, circCUX1 binds to Caspase1 and inhibits its expression, resulting in a decrease in the release of inflammatory factors, thereby developing tolerance to radiotherapy. Our findings indicate that circCUX1 is a potential therapeutic target for radiotherapy tolerance in HPSCC patients.

## Introduction

Head and neck squamous cell carcinoma (HNSCC), including laryngeal cancer, oral cancer, nasopharyngeal cancer, hypopharyngeal cancer, has many primary sites and pathological types and more than 90% of head and neck tumors are squamous cell carcinoma^1^. Patients with early stage HNSCC can obtain good curative effects by surgery or radiotherapy. However, the prognosis of advanced patients is often unsatisfactory^2,3^. Understanding the pathogenesis of HNSCC is crucial for improving the diagnosis and cure rate of patients. Hypopharyngeal squamous cell carcinoma (HPSCC) is one of the most common malignant tumors in otolaryngology head and neck surgery^4^. It has the characteristics of hidden location, strong infiltration, easy submucosal spread, and multicentric growth of the primary lesion. Because there are no obvious symptoms in the early stage, the lack of specific signs and reliable diagnostic measures, most HPSCC patients are already in the advanced stage when they are diagnosed^5^. In addition, chronic inflammation is often a key factor in cancer development. As the head and neck area is prone to exposure to factors causing irritation and inflammation of the squamous epithelium, it might therefore be plausible that chronic inflammation also might be a major cause for the development of HNSCC^6,7^. there are abundant lymphatic vessels in the laryngopharyngeal area, which are prone to cervical lymph node metastasis and extracapsular spread, and invade surrounding tissues and organs, such as carotid arteries and other important blood vessels^8^. HPSCC is one of the worst prognostic malignant tumors. Therefore, it is an urgent issue to propose new diagnostic methods and less toxic and side effects treatment methods based on molecular biology.

Circular RNAs (circRNAs) are a special type of RNA molecule, mostly circRNA molecules without 5′ and 3′ ends formed by reverse splicing of more than one exon, which are abundant in eukaryotic cells^9^. CircRNAs have high degree of conservation and tissue specificity^10^. At present, the mechanism of regulating the expression of circRNA is not completely clear. Studies have found that the production and function of non-coding RNAs such as microRNA (miRNA), long-chain non-coding RNA (lncRNA), and circRNA (circRNA) are regulated by N6-methyladenosine (m6A) modification^11^. M6A refers to the methylation modification that occurs on the sixth nitrogen atom of adenine, and it is widely present in the RNA of many eukaryotes. Current research shows that m6A-related proteins mainly include three types of methyltransferases that catalyze the formation of m6A, demethylases that remove m6A modifications, and methyl-binding proteins that recognize m6A modifications^12^. Recent studies have found that there are also m6A modifications on circRNA, which are jointly regulated by the demethylase FTO and the methyltransferase METTL3^13^. In the tumor tissues of patients with primary non-small cell lung cancer, the positive expression rate of METTL3 is higher than that of normal tissues around the cancer, and the level of METTL3 is associated with the patient’s tumor size, depth of invasion, and lymph node metastasis^14^. After the use of small interference RNA interference technology to reduce the level of METTL3 in lung cancer cells, the level of m6A modification on RNA is reduced, and malignant phenotypes such as cell proliferation, migration, and invasion are inhibited^14^. However, the expression and function of circRNA regulated by m6A are still unclear in HNSCC.

Endogenous circRNA participates in gene expression regulation through different mechanisms and performs different functions^15^. CircRNA is more stable than mRNA, miRNA, and long non-coding RNA (lncRNA) in exosomes, plasma, urine, and saliva^16^. Therefore, circRNA is expected to become a biomarker for disease diagnosis. Studies have found that circRNA CDR1as promote the growth and glucose metabolism of nasopharyngeal carcinoma cells by sponging miR-7^17,18^. The latest study found that circRNA 0000285 increased significantly in the serum and tissues of patients with nasopharyngeal carcinoma, and affected the sensitivity of radiotherapy in patients with nasopharyngeal carcinoma^19^. CircRNA microarray analysis of HPSCC obtained 2392 differential circRNA molecules^20^. However, the role of these circRNAs in HPSCC is not fully clear.

In this study, we demonstrated that circCUX1 was upregulated in HPSCC patients who were resistant to radiotherapy and predicted a poor survival outcome. We further found that METTL3-mediated the m6A methylation of circCUX1 and stabilized its expression. Knockdown circCUX1 promoted the sensitivity of hypopharyngeal cancer cells to radiotherapy. CircCUX1 bound to caspase1 and inhibits its expression, resulting in a decrease in the release of inflammatory factors, thereby developing tolerance to radiotherapy. Our findings indicate that circCUX1 is a potential therapeutic target for radiotherapy tolerance in HPSCC patients.

## Materials and methods

### Tumor sample collection

This study was approved by the ethics committee of Xiangya Hospital of Central South University. Before the operation, written informed consent was signed by the patient. Inclusion criteria: (1) First diagnosed in our hospital and confirmed pathologically as HPSCC; after diagnosis, radiotherapy was performed (2) Re-examination in our hospital 3 months after the end of radiotherapy, the imaging data was complete; At 6 months, the cervical lymph nodes did not completely disappear. The neck lymph node dissection specimens were performed, and the patient was pathologically diagnosed as a head and neck tumor and neck metastasis. From 2015 to 2019, human tumor tissues were collected from 78 patients with HPSCC collected from our hospital, including 68 males and 10 females, aged 28–76 years, with a median age of 51 years. And 60 cases of adjacent tissues during the same period were used as the control group. According to WHO’s radiotherapy response assessment criteria for solid tumors^21,22^, 3 months after the end of radiotherapy, patients with HPSCC were divided into radiotherapy-sensitive group (*N* = 40) and radiotherapy-resistant group (*N* = 38). All patients receive regular follow-up. The overall survival (OS) time is from the date of surgery to the date of death or the last follow-up of the survivor. The disease-free survival (DFS) is the survival time from the first radiotherapy to the tumor recurrence or death.

### Cell culture

The head and neck tumor cell lines SCC-4, SCC-9, SCC-15, CLA-27, SAS, Tca-8113, and Fadu cell lines, and human oral keratinocyte (HOK) cells were purchased from the American Type Culture Collection (ATCC). All cells were cultured in RPMI-1640 containing 10% fetal bovine serum medium (Invitrogen, Carlsbad, USA). All cells were cultured in a humidified incubator at 37 °C and 5% carbon dioxide.

### RNA-seq analysis

**Fig. 1:**
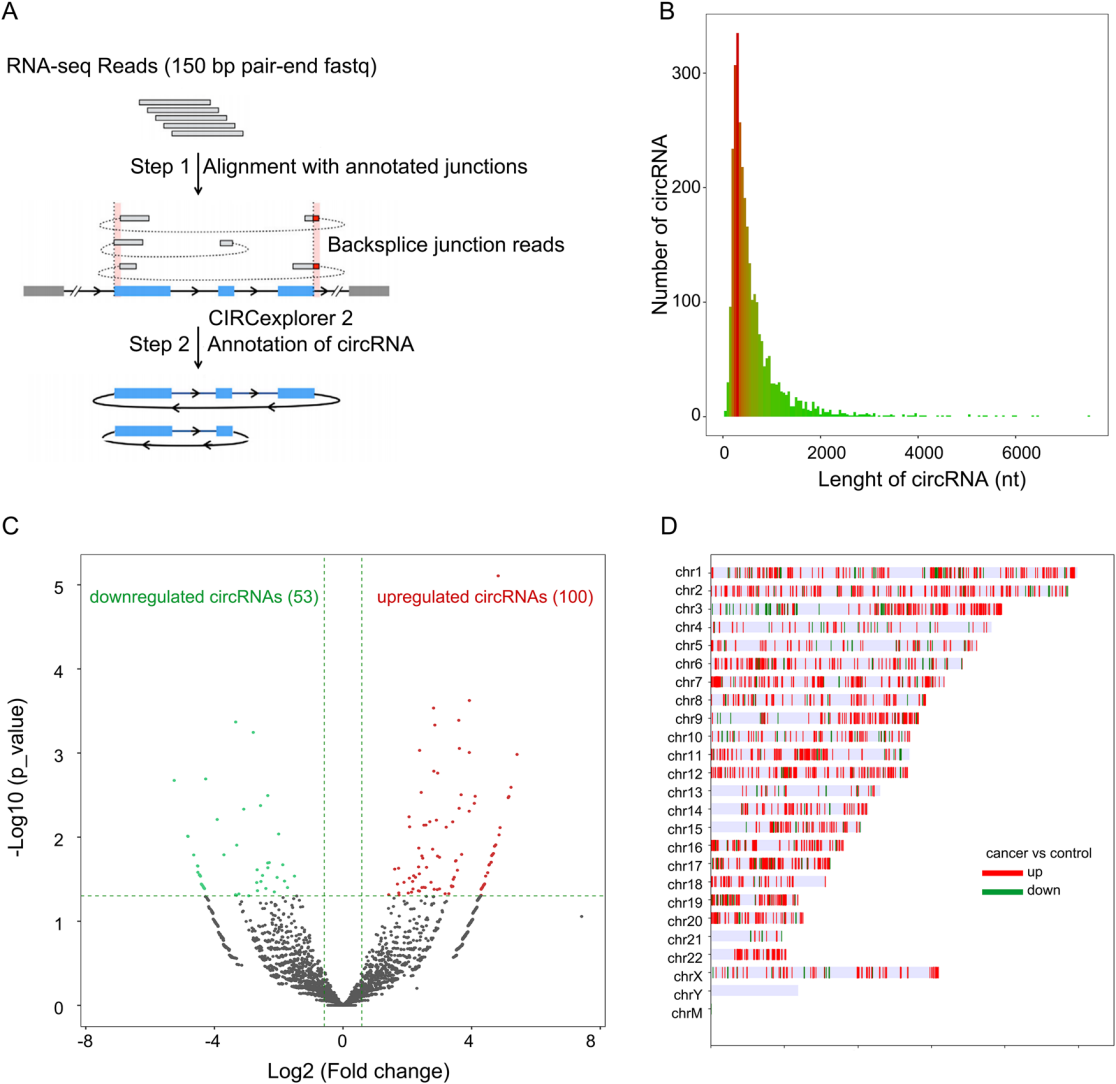
RNA sequencing reveals the dysregulated circRNAs in HPSCC. **A** Flowchart illustrating the RNA sequencing and circRNA annotation. **B** circRNAs length distribution. **C** Volcano plot showed the downregulated circRNA and upregulated circRNA. **D** Downregulated and upregulated circRNA distribution in chromosome. chM chromosome mitochondria.

Five pairs of HPSCC tumor samples and matched paracancerous samples (included 3 pairs of sensitive specimens and 2 pairs of resistant specimens) were randomly selected for RNA sequencing analysis, which was completed by Aksomics (Shanghai, China). NanoDrop ND-1000 was used to determine the RNA concentration. One to two micrograms of total RNA from each sample was used to construct the RNA sequencing library. Briefly, after the total RNA sample is enriched with oligo dT (rRNA removal), using KAPA Stranded RNA-Seq Library Prep Kit (Illumina) constructs the library. The double-stranded cDNA synthesis in the library construction process using the dUTP method combined with the subsequent high-fidelity PCR polymerase to make the final RNA sequencing library chain specific. The constructed library was identified by Agilent 2100 Bioanalyzer for library quality, and the library was quantified by qPCR method. The mixed library of different samples is sequenced using the Illumina HiSeq 4000 sequencer. Solexa pipeline version 1.8 (Off-Line Base Caller software, version 1.8) software was used for image processing and base identification. FastQC software evaluates the sequencing quality of reads after removing adapters. R software Ballgown was used to calculate FPKM at gene level and transcript level, and calculate the difference in gene level and transcript level expression respectively, and screen the differentially expressed genes between samples or groups. New gene/transcript prediction is assembled by StringTie for each sample, and compared with official annotation information, obtained through Ballgown calculation. CircRNAs were compared to the reference genome by STAR software, and CIRCexplorer2 was used for Backsplice junction reads detection, reads count statistics, and R software edge R was used for differential expression calculation. The sequencing flowchart is shown in Fig. 1A. The threshold was set to be 1.5-fold difference, *p* value ≤0.05 and intra-group CPM average ≥100 to screen for differentially expressed circRNA.

### RNA quantitative real-time polymerase chain reaction

TRIzol kit was used to extract total RNA from tissues and cells. UV spectrophotometer was used to detect RNA quality. QuantiTect reverse transcription kit (Cat No. 205313, Qiagen, USA) was used to reverse transcription to cDNA. Real-time polymerase chain reaction (RT-PCR) was performed using SYBR Green Master Mix (Bio-Rad, USA) on ABI7900HT (Applied Biosystems, CA, USA). The reaction conditions are: 95 °C for 10 s; 95 °C for 5 s, 60 °C for 30 s, 72 °C for 30 s, 40 cycles. β-actin was used as an internal reference gene. The primer sequences are listed in supplementary Table 1.

### Actinomycin D and RNase R treatment

Fadu cells were seeded into a six-well plate. After 24 h, when reached 70% confluence, the cells were treated with 5 μg/ml actinomycin and collected at the designated time point. For RNase R treatment, total RNA (2 μg) and 3 U/μg RNase R (Epicenter Technologies, Madison, WI, USA) were incubated at 37 °C for 15 min. After actinomycin D or RNase R treatment, the RNA expression levels of circCUX1 and CUX1 mRNA were analyzed by qRT-PCR.

### RNA fluorescence in situ hybridization (FISH)

The sequence of the oligonucleotide modified probe circCUX1 was synthesized by Sangon Biotech (Shanghai, China). The paraformaldehyde-fixed cell slides were washed in PBS, and treated with RNase R at 37 °C for 15 min, and then fixed again. After dehydration with ethanol, the probes were hybridized overnight at 37 °C in a dark box. The slides were washed twice with 50% formamide for 5 min each. The slides were incubated with Alexa Fluor 488 reagent (Proteintech, Wuhan, China) for 30 min, and sealed with a parafilm containing DAPI. A fluorescence microscope (OLYMPUS, Japan) was used to obtain the pictures. The probe sequence is as follows: circCUX1: 5′-Biotin labeled-UCCCGACACACUCGAGUCUAACUACGUGACUCAUUUCUUCGUUC -3′.

### RNA interference (RNAi) and transfection

The siRNA used to knock out circCUX1 and METTL3 was designed and synthesized from Ribobio (Guangzhou, China). The siRNA target sequence used to construct the lentivirus as: circCUX1 siRNA 1#, TGTGAGGAGTCTAACTAGCACTGAGT; circCUX1 siRNA 2#, GACAAAGAGATTGATGCACTGA; METTL3 siRNA, GCACAUCCUACUCUUGUAATT. Fadu or SCC-9 cells are seeded into a 24-well culture plate at a density of 4 × 10^4^ cells per well to ensure that the cell confluence can reach about 60% during transfection. The medium was replaced with 500 μl fresh complete medium, and added with 6 μl each well of the lentiviral particle stock solution and negative control virus to the 24-well culture plate, accompanied with 0.5 μl polybrene (final concentration 5 ng/ml), The cells were cultured in 37 °C incubator for 48 h, and collected for subsequent experiments.

### ELIZA

After the cells are transfected, the cell culture supernatant is collected. The human IL-1β and IL-18 kit (cat no. KE00021, KE00025; Proteintech Group, Wuhan, China) were used to analyze the IL-1β and IL-18 concentrations using follow the manufacturer’s instructions.

### Cell growth inhibition assay

Fadu or SCC-9 cells were transfected with lentiviruses for 48 h and then seeded into a 6-well (5 × 10^5^ per well) plate and cultured overnight. A medical electron linear accelerator was used for X-ray irradiation (distance 100 cm, irradiation area 20 cm × 20 cm, single irradiation, irradiation dose of 0, 1, 2, 4, 6 Gy). After irradiation, cells were continually cultured for 24 h. The cell survival rate was detected by Cell Counting Kit 8 (CCK-8) (Abcam, China) according to the manufacturer’s instructions. CCK-8 allows very convenient assays by utilizing highly water-soluble tetrazolium salt. WST-8 [2-(2-methoxy-4-nitrophenyl)-3-(4-nitrophenyl)-5-(2,4-disulfophenyl)-2H-tetrazolium, monosodium salt] produces a water-soluble formazan dye upon reduction in the presence of an electron mediator. CCK-8 allows sensitive colorimetric assays for the determination of the number of viable cells in cell proliferation assays. The median lethal dose (LD50) was calculated.

### RNA pull-down

The biotin-labeled circCUX1 probe was mixed with total cell protein and incubated for 24 h at 4 °C to make circCUX1 and protein form a circCUX1-protein complex, and then the magnetic beads (MEGA clear Kit, Ambion) were used to adsorb the complex. The protein in the capture complex was analyzed by Western blotting, using sodium dodecyl sulfate (SDS)-polyacrylamide gel (PAGE) electrophoresis. The proteins were transferred on polyvinylidene fluoride (PVDF) membrane. The membrane was incubated with 5% non-fat milk for 1 h at 37 °C and incubated with a primary antibody METTL3 (diluted 1:1000, Abcam) overnight at 4 °C. After washed with PBS, the membrane was incubated with the secondary antibody for 2 h at room temperature. A SuperSignal West Atto Ultimate Sensitivity Substrate (cat no. A38555, ThermoFisher, USA) was used to protein blot detection in the gel imaging system.

### RNA-binding protein immunoprecipitation (RIP)

A Magna RIPTM RNA-Binding Protein Immunoprecipitation Kit from Millipore was used for RNA immunoprecipitation in accordance with the kit instructions. Briefly, after 48 h transfection, Fadu cells were harvested and lysed with RIP lysis buffer on ice for 10 min. After centrifugation, the supernatant was incubated with 30 μl Protein-A/G magnetic beads (Roche, USA) and antibody (METTL3, 10 μg, Abcam; METTL14, 10 μg, Abcam) at 4 °C overnight. The complex was then centrifuged and then washed 3 times with washing buffer. Immunoprecipitated RNA was used in qRT-PCR analysis.

### Luciferase reporter gene determination

The wild type (WT) or mutant (Mut) Caspase 1 cDNA fragments of the target binding site predicted by circCUX1 were inserted into the luciferase report gene pGL3 (Promega) to construct a recombinant vector. Fadu cells were co-transfected with circCUX1 (800 ng) or circCUX1 siRNA (20 pmol) and WT or Mut Caspase 1 (20 pmol) in a 24-well culture plate (1 × 10^5^ cells per well). The transfection process was carried out using Lipofectamine 2000 system. After 48 h of transfection, the cells were collected, and the luciferase activity was detected by a Thermo Scientific Vanquish system (Thermo Scientific, USA).

### Statistical analysis

SPSS 16. 0 software was used to carry on statistical analysis to the data. All experimental data were performed independently three times, and the data was expressed as mean ± standard deviation (mean ± SD). The obtained data were compared using one-way analysis of variance and Bonferroni’s post-correction Student’s *t* test. The difference was statistically significant with *P* < 0.05.

## Results

### Analysis of dysregulated circRNA in HPSCC tissue

We performed high-throughput RNA sequencing in five pairs of HPSCC and matched adjacent non-tumor tissue samples. The experimental process is shown in Fig. 1A. The length of circRNA expressed in HPSCC tissues is mainly within 2000 nt (Fig. 1B). Screening by the following criteria: RNA expression was upregulated or downregulated, and average normalized fold change ≥2. We found 53 downregulated circRNAs and 100 upregulated circRNAs (Fig. 1C); Table 1 lists the top ten upregulated circRNAs. Except for Y chromosome and mitochondria, dysregulated circRNAs were found in all other chromosome (Fig. 1D).

**Table 1:** Top ten upregulated circRNAs in HPSCC tissues.

circRNA	Locus	Gene Name	Length	Fold Change	*P* value	*Q* value
hsa_circ_0114428	chr1:91403041-91447927:−	ZNF644	3705	42.57	0.00103	0.28247
hsa_circ_0081607	chr7:101559394-101838883:+	CUX1	1192	37.39	0.00255	0.46150
hsa_circ_0008255	chr16:16200594-16205439:+	ABCC1	344	35.72	0.00328	0.46150
hsa_circ_0008992	chr11:77090285-77091039:−	PAK1	249	35.20	0.00339	0.46150
hsa_circ_0006051	chr22:25771779-25771975:−	LRP5L	196	29.53	0.00770	0.62274
hsa_circ_0112397	chr1:232649602-232651354:−	SIPA1L2	1752	28.90	0.00940	0.71638
hsa_circ_0057608	chr2:197777605-197786910:−	PGAP1	586	26.91	0.01235	0.72194
hsa_circ_0008912	chr12:72020039-72022817:−	ZFC3H1	491	26.19	0.01284	0.72194
hsa_circ_0006358	chr4:169812072-169825057:+	PALLD	607	25.12	0.01609	0.72194
hsa_circ_0095454	chr11:16339991-16362798:−	SOX6	449	25.09	0.01612	0.72194

**Fig. 2:**
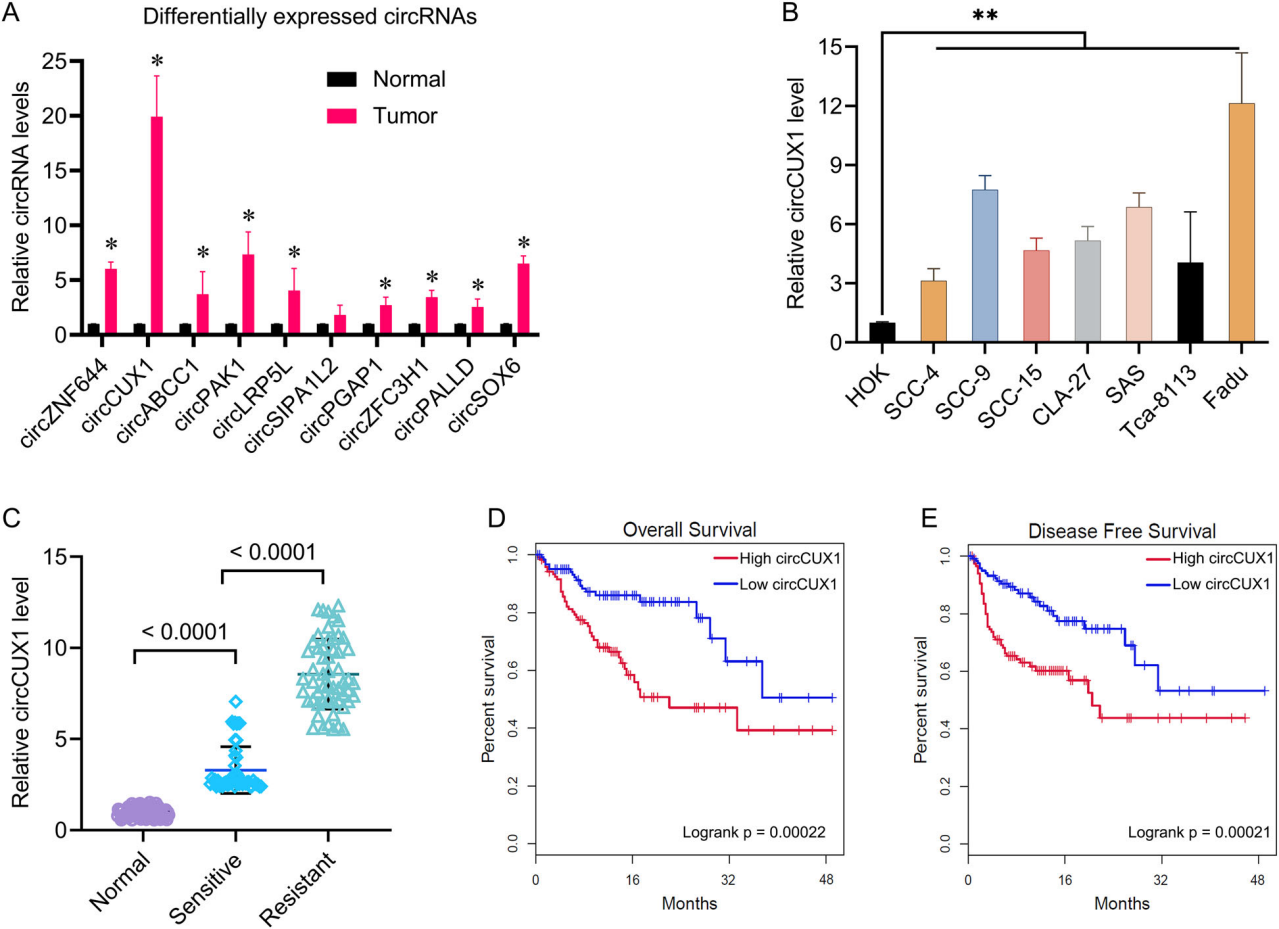
CircCUX1 was upregulated in HPSCC with radiotherapy resistance. **A** qRT-PCR analysis of upregulated circRNAs expression in ten HPSCC tissues and matched adjacent normal tissue. *p < 0.05. **B** qRT-PCR analysis of circCUX1 expression in HOK and seven head and neck tumor cell lines. **C** qRT-PCR analysis of circCUX1 expression in radiosensitive (*N* = 40) and radioresistant (*N* = 38) HPSCC tissues and adjacent normal tissue (*N* = 60). **D** Kaplan–Meier analysis of overall survival (OS) in HPSCC patients with low vs. high expression of circCUX1. **E** Kaplan–Meier analysis of disease-free survival in HPSCC patients with low vs. high expression of circCUX1.

We used qPCR to verify the expression of differential circRNA in Table 1 in ten pairs of HPSCC and matched adjacent non-tumor tissue samples, including four pairs of sensitive specimens and six pairs of resistant specimens that was different from the panel of five matched pairs of clinical specimens in initial screening. We found that except for the expression of circSIPA1L2, the expression of other circRNAs in tumor tissues was significantly higher than that in adjacent tissues, especially the expression of circCUX1 (Fig. 2A). Therefore, we choose circCUX1 (circRNA_0081609, hg 19, chr7: 101713618–101848450, exon 4 – exon 20) for further analysis. We tested the expression of circCUX1 in HOK and seven head and neck tumor cell lines. Compared with HOK cells, the expression of circCUX1 in head and neck tumor cell lines was significantly increased, especially in the hypopharyngeal carcinoma cell line Fadu (Fig. 2B).

To study the clinical significance of circCUX1 in hypopharyngeal cancer patients, a cohort of 78 hypopharyngeal cancer patients with survival data was included. Since radiotherapy is an important treatment for patients with head and neck tumors, and radiotherapy tolerance is an important prognostic predictor, we further investigated whether the expression of circCUX1 is related to radiotherapy tolerance in patients with hypopharyngeal carcinoma. The 78 patients included 38 radiotherapy-resistant patients and 40 radiotherapy-sensitive patients, and 60 adjacent non-tumor tissues were collected as controls. We found that compared with the control group, the expression of circCUX1 was significantly upregulated in cancer tissues, especially in patients with radiotherapy tolerance (Fig. 2C). In addition, circCUX1 was induced by irradiation in Fadu and SCC-9 cells (Supplementary Fig. 1). We next used the mean value of circCUX1 expression as the cutoff value to divide the patients into high circCUX1 group and low circCUX1 group. Through Kaplan–Meier analysis, we found that high expression of circCUX1 indicated poor OS and poor DFS (Fig. 2D, E). We further analyzed the correlation between the expression of circCUX1 and the clinical characteristics of patients with HPSCC. As shown in Table 2, the expression of circCUX1 was significantly correlated with primary tumor size (*p* < 0.001), lymph node metastasis (*p* = 0.0121), distant metastasis (*p* = 0.0394), and TNM stage (*p* = 0.0219). There was no significant correlation between the expression of circCUX1 and age (*p* = 0.2643). Univariate and multivariate Cox regression analysis found that circCUX1 was an independent factor affecting the survival of patients with HPSCC (Univariate analysis: HR, 3.87; 95% CI, 1.94–6.24, *p* = 0.0063; multivariate analysis: HR, 2.83; 95% CI, 1.74–5.02, *p* = 0.0167) (Tables 2, 3, and 4), suggesting that circCUX1 can be used as a biomarker for HPSCC.

**Table 2:** Association of HPSCC characteristics and circCUX1 expression.

	High circCUX1	Low circCUX1	*P* value
*N*, %	45 (57.7)	33 (42.3)	
Age, year, mean (SD)	48.8 (15.3)	54.3 (10.4)	0.2643
Tumor size, cm, mean (SD)	2.6 (1.4)	3.8 (1.2)	<0.001
Lymphatic metastasis, *N*			0.0121
*N*0	18	23	
*N*1–3	27	10	
Distant metastasis, *N*			0.0394
No	22	24	
Yes	23	9	
TNM stage, *N*			0.0219
I or II	15	20	
III or IV	30	13

**Table 3:** Univariate analysis of prognostic factors of HPSCC.

Variable	Hazard ratio (95% CI)	*p* value
Tumor size (≥3 vs. <3)	2.24 (1.66–5.42)	0.0213
Lymphatic metastasis (*N*1–3 vs. *N*0)	3.14 (2.24–5.86)	0.0162
Distant metastasis (Yes vs. No)	5.21 (3.53–7.72)	0.0031
TNM stage ((III–IV vs. I/II)	4.56 (2.67–8.25)	0.0056
circCUX1 (High vs. Low)	3.87 (1.94–6.24)	0.0063

**Table 4:** Multivariate analysis of independent prognostic factors of HPSCC.

Variable	Hazard ratio (95% CI)	*p* value
Tumor size (≥3 vs. <3)	1.97 (1.27–3.57)	0.0352
Lymphatic metastasis (*N*1–3 vs. *N*0)	2.79 (1.65–4.68)	0.0265
Distant metastasis (Yes vs. No)	4.35 (2.04–6.87)	0.0125
TNM stage ((III/IV vs. I/II)	3.16 (2.07–6.15)	0.0086
circCUX1 (High vs. Low)	2.83 (1.74–5.02)	0.0167

### Characterization of circCUX1 in hypopharyngeal carcinoma cells

**Fig. 3:**
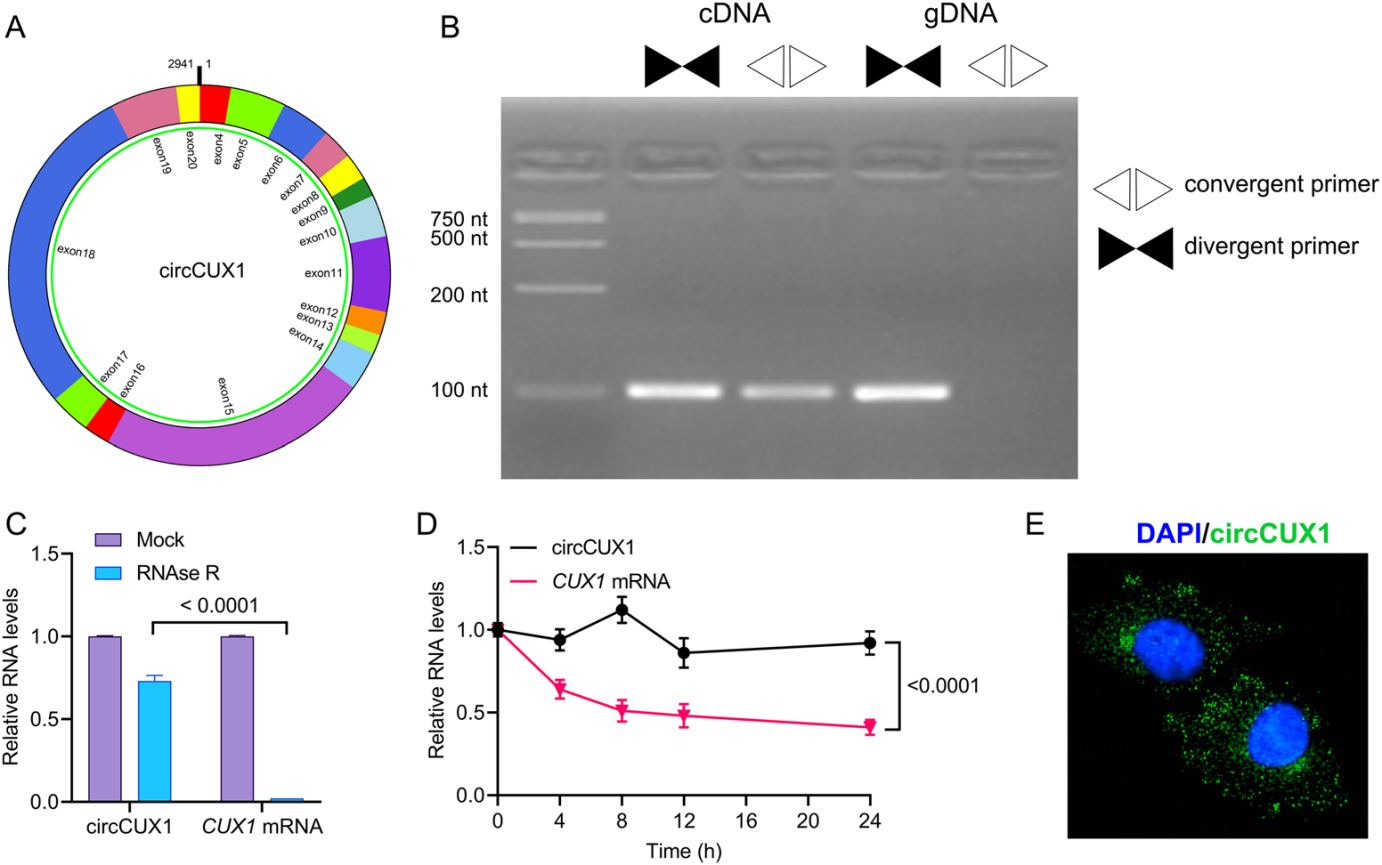
Characterization of circCUX1 in HPSCC. **A** The circCUX1 structure. **B** qRT-PCR products with divergent primers showing circularization of circCUX1. cDNA complementary DNA, gDNA genomic DNA. **C** qRT-PCR analysis for the expression of circCUX1 and CUX1 mRNA after treatment with RNase R in Fadu cells. **D** qRT-PCR analysis for the expression of circCUX1 and CUX1 mRNAs after treatment with Actinomycin D at the indicated time points in Fadu cells. **E** RNA fluorescence in situ hybridization for circCUX1. Nuclei were stained with DAPI.

CircCUX1 is derived from exons 4 to 20 (hg 19, chr7: 101713618–101848450, GRCh37/hg 19) in the cut-like homeobox 1 (CUX1) locus, which has been described in neuroblastoma by Li et al.^23^. We aim to study the characterization of circCUX1 in HPSCC. Divergent and convergent primers were used to check the specificity of the primers and the correctness of the circularization site (5′ exon 20 to 3′exon 4) by RT-PCR (Fig. 3A, B). RNase R-resistant exonuclease digestion confirmed that circCUX1 has a circRNA structure (Fig. 3C). Actinomycin D is a transcription inhibitor that can be used to analyze the half-life of RNA. After actinomycin D treatment, qRT-PCR analysis showed that the half-life of circCUX1 exceeded 24 h, while the half-life of linear transcript approximately was 8 h (Fig. 3D), indicating that circCUX1 is more stable in hypopharyngeal cancer cells. Further, fluorescence in situ hybridization (FISH) revealed that circCUX1 was mainly located in the cytoplasm (Fig. 3E). Together, these results reveal that circCUX1 is a rich and stable circRNA expressed in hypopharyngeal cancer cells.

### METTL3-mediated m6A modification stabilizes the expression of circCUX1

**Fig. 4:**
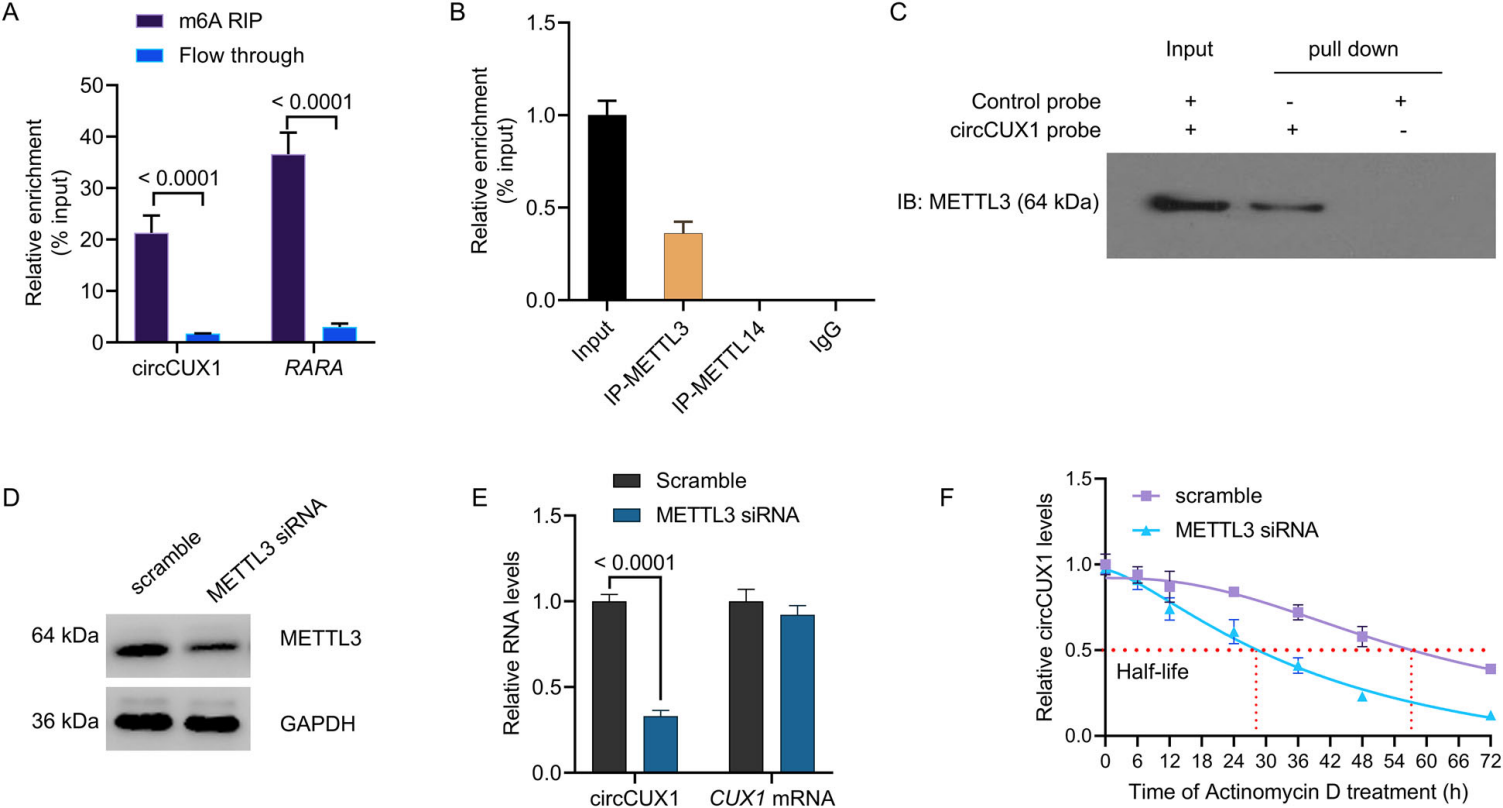
METTL3 promotes m6A-methylated circCUX1. **A** RIP assay showing that circCUX1 was highly recruited in m6A precipitated fraction in Fadu cells. **B** RIP assays showing the association of METTL3 and METTL14 with circCUX1 in Fadu cells. Relative enrichment representing RNA levels associated with METTL3 and METTL14 relative to an input control. IgG antibody served as a control. **C** The circCUX1-protein complex pulled down by circCUX1 junction probe with protein extracts from Fadu cells. Immunoblot analysis of METTL3 after pull-down assay showing its specific association with circCUX1. **D** Western blot analysis for the expression of METTL3 after METTL3 siRNA transfection in Fadu cells. **E** qRT-PCR analysis for the expression of circCUX1 and CUX1 mRNA after METTL3 siRNA transfection in Fadu cells. **F** qRT-PCR analysis for the expression of circCUX1 after treatment with Actinomycin D at the indicated time points in Fadu cells.

The recent researches show the important role of m6A epigenetic modification in tumor radiotherapy. We want to know whether circCUX1 contains m6A methylation. Through methylated RNA immunoprecipitation (MeRIP) analysis, we precipitated a known m6A-containing RNAs, RARA, and circCUX1 also contains abundant m6A methylation (Fig. 4A), confirming the m6A methylation in circCUX1. METTL3 is a predominant methyltransferase for m6A modification and plays an oncogenic role in tumor formation and progression^24^. We performed RNA-binding protein immunoprecipitation (RIP) analysis and proved that compared with the control IgG, METTL3 antibody-precipitated complexes enriched the expression of circCUX1, while METTL14 could not enrich circCUX1 (Fig. 4B). We performed an RNA pull-down experiment to further verify the combination of METTL3 and circCUX1 (Fig. 4C). In addition, we want to know that the m6A methyltransferase METTL3 whether affect circCUX1 activity. Silencing of METTL3 significantly inhibited the expression of circCUX1 (Fig. 4D, E), and shortened the half-life of circCUX1 RNA compared with scramble (METTL3 siRNA: 28 h vs. scramble: 57 h, Fig. 4F). These results were also confirmed in SCC-9 cells (Supplementary Fig. 2). The above results suggest that METTL3 stabilizes the expression of circCUX1 through m6A methylation modification.

### Silencing circCUX1 promotes the sensitivity of hypopharyngeal cancer cells to radiotherapy

**Fig. 5:**
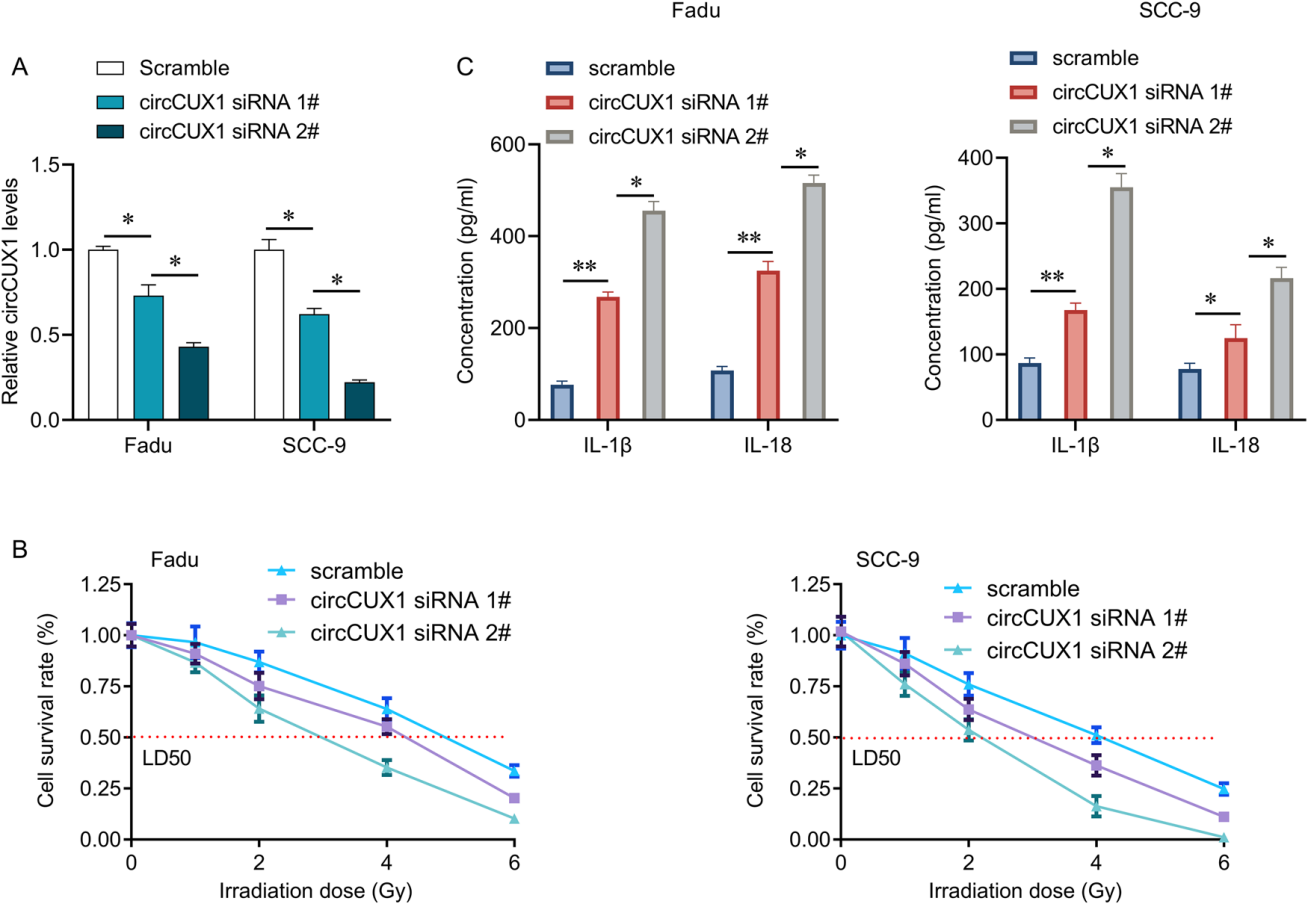
CircCUX1 knockdown promotes radiosensitive of HPSCC. **A** qRT-PCR analysis for the expression of circCUX1 after siRNA transfection in Fadu and SCC-9 cells. **B** CCK-8 assay for the cell survival rate after siRNA transfection and irradiation in Fadu and SCC-9 cells. **C** The concentration of IL-1β and IL-18 was determined by ELIZA after siRNA transfection and irradiation in Fadu and SCC-9 cells. *p < 0.05, **p < 0.01.

We knocked down the expression of circCUX1 in Fadu and SCC-9 cells by siRNA transfection. We found that siRNA 2# transfection had higher efficiency in inhibiting circCUX1 expression than siRNA 1# transfection (Fig. 5A), while both siRNA 1# and siRNA 2# transfection did not alter the expression of CUX1 mRNA (Supplementary Fig. 3). Our results showed that circCUX1 knockdown significantly inhibited the cell viability, and increased the release of inflammatory factors IL-1β and IL-18 in Fadu and SCC-9 cells without irradiation exposure (Supplementary Fig. 4). We then studied the role of circCUX1 in radiotherapy by determining the cell viability through a CCK-8 experiment. The results showed that circCUX1 knockdown significantly reduced the LD50 of Fadu and SCC-9 cells, and the reduction of LD50 was positively associated with the decrease of circCUX1 levels (SCC-9 cells: LD50 in scramble: 4.1 Gy, LD50 in siRNA 1#: 3.1 Gy vs. LD50 in siRNA 2#: 2.2 Gy, *p* < 0.05; Fadu cells: LD50 in scramble: 4.9 Gy, LD50 in siRNA 1#: 4.25 Gy vs. LD50 in siRNA 2#: 2.95 Gy, *p* < 0.05), suggesting that circCUX1 knockdown promoted the sensitivity of cells to radiotherapy (Fig. 5B). In addition, circCUX1 knockdown also significantly increased the release of inflammatory factors IL-1β and IL-18, and the levels of IL-1β and IL-18 in siRNA 2# group were higher than in siRNA 1# group (Fig. 5C).

### CircCUX1 regulates Caspase 1 mRNA

**Fig. 6:**
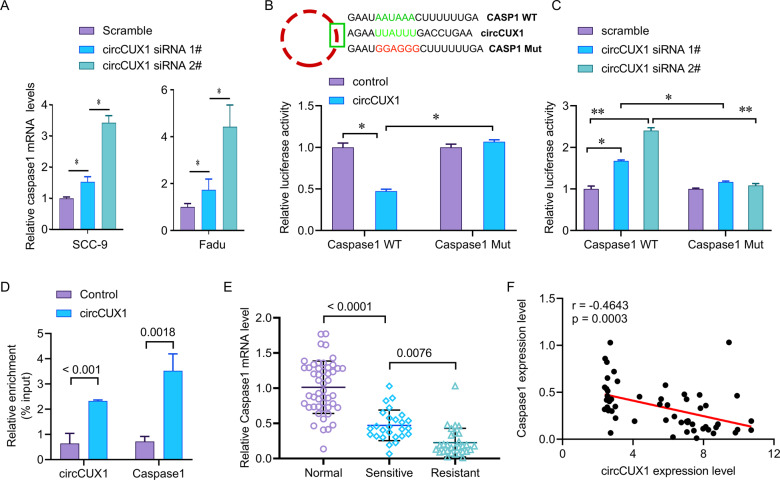
CircCUX1 inhibits caspase1 mRNA. **A** qRT-PCR analysis for the expression of caspase1 after siRNA transfection in Fadu cells. **B** Relative luciferase activity of luciferase reporter gene with caspase1-WT or caspase1-Mut in control and circCUX1-overexpression Fadu cells. The wild type motifs were indicated by green and the mutant motif was indicated by red. **C** Relative luciferase activity of luciferase reporter gene with caspase1-WT or caspase1-Mut in control and circCUX1 knockdown Fadu cells. **D** Relative enrichment representing caspase1 and circCUX1 RNA levels associated with circCUX1 junction compared to control. **E** qRT-PCR analysis of caspase1 expression in radiosensitive (*N* = 40) and radioresistant (*N* = 38) HPSCC tissues and adjacent normal tissue (*N* = 60). **F** circCUX1 expression showing negatively correlated with caspase1 expression in HPSCC patients (*N* = 78). *p < 0.05, **p < 0.01.

Studies have shown that Caspase-1 can directly cleave IL-1β precursor (pro-IL-1β) and IL-18 precursor (pro-IL-18) to make them active IL-1β and IL-18, and release them to extracellular area becomes an inducer of inflammatory response and participates in the progression of inflammation-related tumors. Knockdown of circCUX1 increased the release of IL-1β and IL-18. We want to know whether circCUX1 can regulate the expression of caspase 1. We confirmed that circCUX1 knockdown can increase the expression of caspase 1 (Fig. 6A). AREsite analysis (http://rna.tbi.univie.ac.at/AREsite2) and BLAST analysis suggested that the UUAUUU site inside of circCUX1 could directly bind to the 3′UTR of caspase 1 with AAUAAA motif. Therefore, we further investigated whether circCUX1 is essential for the stability of caspase1 mRNA. We constructed WT caspase1–3′UTR (caspase1-WT) or Mut 3′UTR (caspase1-Mut). Overexpression of circCUX1 significantly inhibited the activity of WT caspase1–3′UTR luciferase reporter gene, but did not affect the activity of Mut caspase1–3′UTR luciferase reporter gene (Fig. 6B). In addition, knockdown of circCUX1 greatly increased luciferase activity of WT caspase 1 (Fig. 6C), but not caspase1-Mut. Our RNA pull-down analysis also confirmed the interaction between circCUX1 and caspase1 (Fig. 6D).

To further reveal the clinical significance of circCUX1 regulating caspase 1 in hypopharyngeal carcinoma, we checked the expression level of caspase1 and analyzed the correlation between caspase1 and circCUX1. We found that compared with normal tissues adjacent to cancer, caspase 1 was significantly reduced in hypopharyngeal cancer tissues, especially in radiotherapy-resistant tissues (Fig. 6E). It is worth noting that the expression of caspase 1 in hypopharyngeal carcinoma tissue was negatively correlated with the expression of circCUX1 (*r* = -0.4643, *p* = 0.0003, *N* = 78, Fig. 6F).

### CircCUX1 promotes radiotherapy resistance of hypopharyngeal carcinoma through caspase 1

**Fig. 7:**
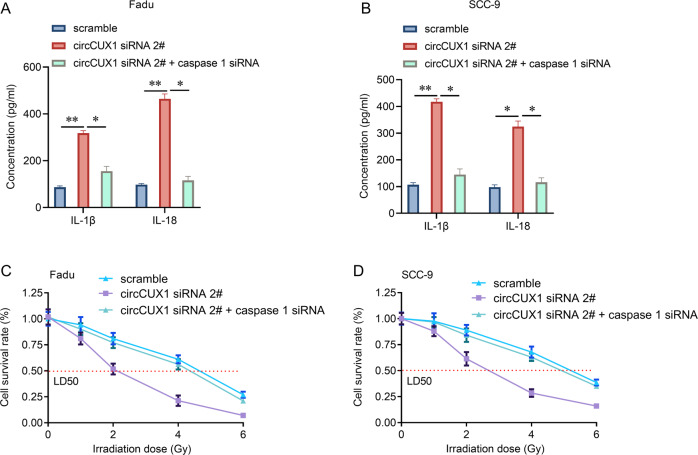
CircCUX1 confers radioresistance of HPSCC through caspase1 pathway. **A**, **B** The concentration of IL-1β and IL-18 was determined by ELIZA after siRNA transfection and irradiation in Fadu (**A**) and SCC-9 (**B**) cells. **C**, **D** CCK-8 assay for the cell survival rate after siRNA transfection and irradiation in Fadu (**C**) and SCC-9 (**D**) cells.

Then, we investigated whether circCUX1 depends on the caspase1 pathway in the progression of radiotherapy tolerance in hypopharyngeal carcinoma. Our results showed that knockdown of caspase1 significantly reversed the release of inflammatory factors IL-1β and IL-18 mediated by circCUX1 knockdown (Fig. 7A, B). In addition, knockdown of caspase1 could significantly reverse circCUX1 knockdown-mediated radiotherapy sensitivity (Fig. 7C, D). These data indicate that circCUX1-promoted hypopharyngeal cancer cell radiotherapy resistance depends on the caspase 1 pathway.

## Discussion

In this study, we first time demonstrated the important role of circCUX1 in the radiotherapy resistance of HPSCC. We proved that HPSCC patients with high expression of circCUX1 had a poor prognosis. We observed a significant increase in the expression of circCUX1 in HPSCC tissues and found that circCUX1 might be used as a diagnostic and prognostic indicator for patients with HPSCC resistant to radiotherapy.

In addition, we found that METTL3-mediated the m6A methylation of circCUX1 and stabilized its expression. Importantly, circCUX1 bound to caspase1 mRNA and inhibited its expression, thereby inhibiting caspase1 mediated inflammation. The discovery of circRNA provides new research ideas for the occurrence, development, treatment, and prevention of tumors, and the high tissue specificity and stability of circRNA make it suitable as a new biomarker^25^. Many researchers report that circRNAs are significantly expressed in tumors, and affect the development of tumors^11^. Although many studies have confirmed the biological functions of circRNA in physiological and pathological processes, the specific mechanism of circRNA in HPSCC needs to be further revealed. M6A is the most abundant modified form of eukaryotic mRNAs, which affects almost all stages of mRNA metabolism, including RNA folding, splicing, translation, and decay, and other RNA modifications^13^. M6A not only affects mRNA metabolism, but also participates in the regulation of non-coding RNA^26^. More and more evidence show that the dynamic changes of m6A modification provide a new direction for the formulation of tumor radiotherapy and chemotherapy strategies^27^. M6A methyltransferase [such as METTL3, METTL14, and METTL16] participates in the progression of malignant tumors by read, write, or erase m6A on bound RNA^24^. The latest research also fully proves the important role of m6A epigenetic modification in tumor radiotherapy and chemotherapy. Studies have found that m6A methylation in RNA can regulate the DNA damage response induced by ultraviolet rays, thereby affecting tumor chemotherapy and radiotherapy sensitivity. METTL3 and METTL14 in the m6A methyltransferase component are expected to become new targets for tumor radiotherapy and chemotherapy sensitization, and METTL3 can promote the chemotherapy and radiotherapy sensitivity of pancreatic cancer cells^24^. Our study provides the first evidence that the stable expression of circCUX1 in the cytoplasm is dependent on the METTL3-mediated m6A modification. The above studies emphasize the importance of m6A as a cancer modification mechanism. The dynamic changes of m6A levels have different regulatory functions on cancer cells. By revealing previously unidentified tumor gene regulatory mechanisms, it provides a basis for exploring the pathogenesis of some tumors and seeking new potential therapeutic targets, and provides new ideas for tumor epigenetic modification mechanisms and tumor gene targeted therapy.

CircRNAs exert their biological functions three mechanisms: (1) regulating gene expression, transcription, and splicing levels; (2) translating into protein to play a role; (3) acting as miRNA sponges through their binding sites to regulate the activity of miRNAs on other target genes^28^. For example, circCUX1 played an oncogenic role in neuroblastoma through targeting miR-16-5p/DMRT2 signaling cascade^29^. In addition, circCUX1 binds to EWS RNA-binding protein 1 to facilitate its interaction with MYC-associated zinc finger protein, resulting in neuroblastoma progression^23^. Herein, we found that circCUX1 can bind to caspase 1 mRNA and inhibit its expression. Interestingly, circCUX1 regulates the inflammatory response of tumor cells to radiotherapy through caspase 1. Since Rudolf Virchow first proposed the hypothesis that “cancer originates from chronic inflammation”, more and more studies have confirmed that the occurrence and development of tumors are inseparable from inflammation^30^. Epidemiologic evidence suggests that ~25% of all human cancer worldwide is associated with chronic inflammation, chronic infection, or both^6,31^. Inflammation, chronic inflammation has become the seventh major biological feature of malignant tumors, and such tumors are also called inflammation-related tumors^32^. Studies have shown that many inflammatory cell infiltration and aggregation of inflammatory factors are one of the significant pathological features of head and neck malignant tumors, suggesting that uncontrollable inflammation plays an important role in the occurrence and development of head and neck malignancies^33^. Caspase-1 is the first Cysteinyl aspartate specific proteinase (Caspase) family member found in mammals, also known as Interleukin-1β converting enzyme (ICE)^34^. Recent studies have found that Caspase-1 not only plays a key role in the inflammatory response, but is also closely related to a new type of programmed cell death-pyroptosis^35^. Caspase-1 can participate in the regulation of the tumor inflammatory microenvironment in many ways, and affect the occurrence, development, invasion, and metastasis of tumors^36^. The expression level of Caspase-1 may be related to the degree of inflammation in tumor tissues. Caspase-1 and its mediated pro-inflammatory substances may play a very important role in the occurrence and development of inflammation-related tumors^37,38^. In many chronic inflammation-related tumors, there is usually the activation of inflammasomes^39^. The activated Caspase-1 in this process can directly cleave the IL-1β precursor (pro-IL-1β) and IL-18 precursor (pro-IL-18) makes it become active IL-1β and IL-18, and is released to the outside of the cell to become an inducer of inflammation. As an important inflammation-related molecule, Caspase-1 can participate in the regulation of tumor inflammation microenvironment in a variety of ways. In the process of interaction between malignant tumor cells and their microenvironment among them, Caspase-1 actively induces tumor cell programmed death and anti-tumor immune surveillance^40^.

## Conclusion

We find that circCUX1 is an important oncogenic circRNA, which may be used as a prognostic biomarker for radiotherapy resistance of HPSCC. METTL3-mediated m6A modification plays a key role in stabilizing the expression of circCUX1, thereby inhibiting the expression of caspase 1 and conferring the radiotherapy resistance of HPSCC. Importantly, our findings provide potential therapeutic targets for patients with HPSCC who are resistant to radiotherapy.

## Supplementary information

supplementary figures

supplementary table 1

## Data Availability

The datasets used and/or analyzed during the current study are available from the corresponding author on reasonable request.
