# Supplementary material for: N6-methyladenosine modification of circCUX1 confers radioresistance of hypopharyngeal squamous cell carcinoma through caspase1 pathway
Source: Cell Death Dis. 2021 Mar 19;12(4):298. doi: 10.1038/s41419-021-03558-2 (PMC7979824; doi:10.1038/s41419-021-03558-2)
Supplement: Supplementary file 1 — supplementary figures [file 41419_2021_3558_MOESM1_ESM.pdf]

Supplementary Figures

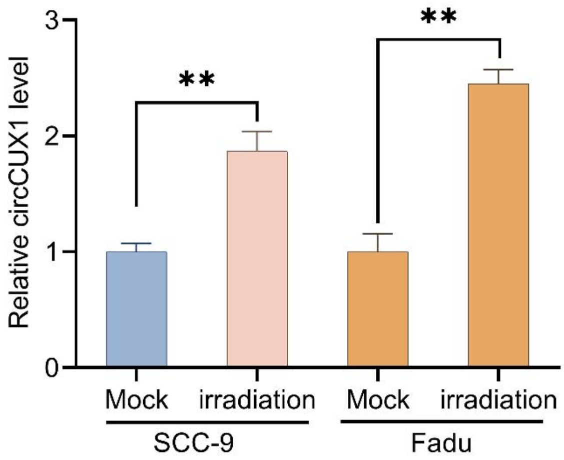

Supplementary Figure 1 circCUX1 was induced by irradiation. qRT–PCR analysis for the expression of circCUX1 in Fadu and SCC-9 cells after irradiation exposure.

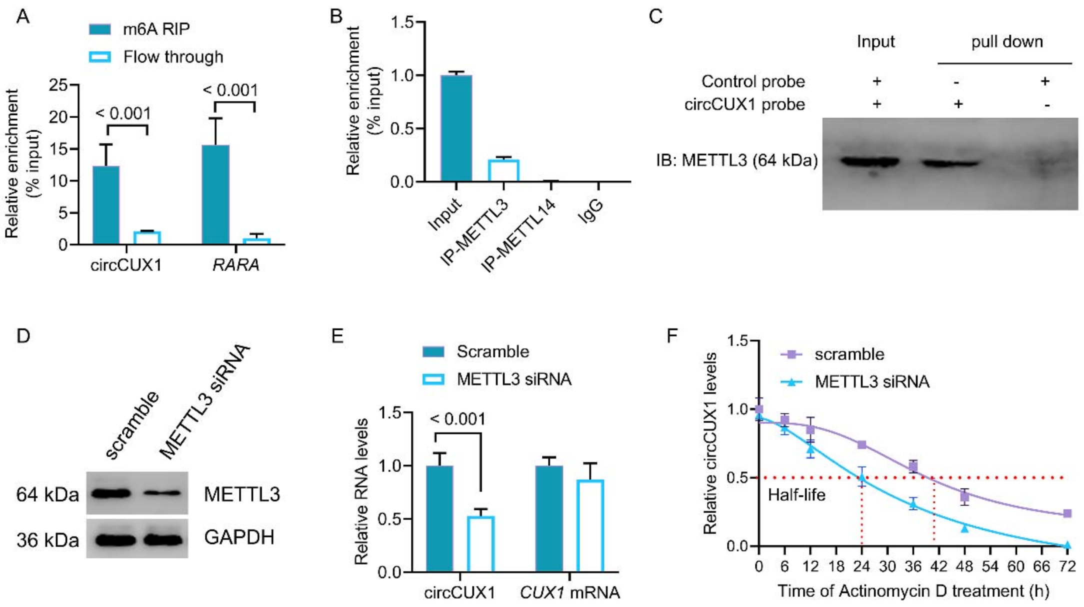

Supplementary Figure 2 METTL3 promotes m6A-methylated circCUX1. (A) RIP assay showing that circCUX1 was highly recruited in m6A precipitated fraction. (B) RIP assays showing the association of METTL3 and METTL14 with circCUX1. Relative enrichment representing RNA levels associated with METTL3 and METTL14 relative to an input control. IgG antibody served as a control. (C) The circCUX1-protein complex pulled down by circCUX1 junction probe with protein extracts from SCC-9 cells. Immunoblot analysis of METTL3 after pulldown assay showing its specific association with circCUX1. (D) Western blot analysis for the expression of METTL3 after METTL3 siRNA transfection in SCC-9 cells. (E) qRT–PCR analysis for the expression of circCUX1 and CUX1 mRNA after METTL3 siRNA transfection in SCC-9 cells. (F) qRT–PCR analysis for the expression of circCUX1 after treatment with Actinomycin D at the indicated time points in SCC-9 cells.

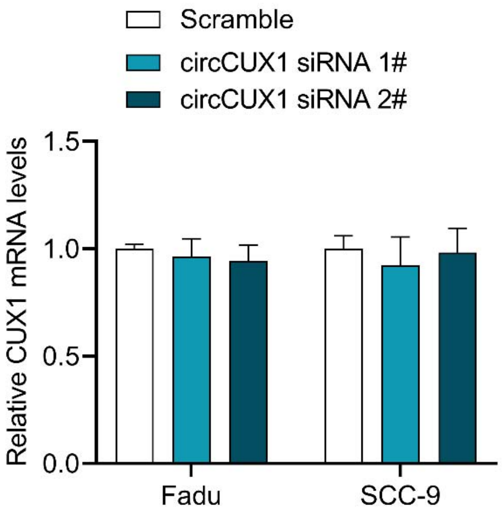

Supplementary Figure 3 The effects of circCUX1 on cell viability and inflammatory factors release. (A) CCK8 assay was performed to measure the cell viability of Fadu and SCC-9 cell after siRNA transfection without irradiation. (B) The concentration of IL-1β and IL-18 was determined by ELISA in Fadu and SCC-9 cells after siRNA transfection without irradiation.

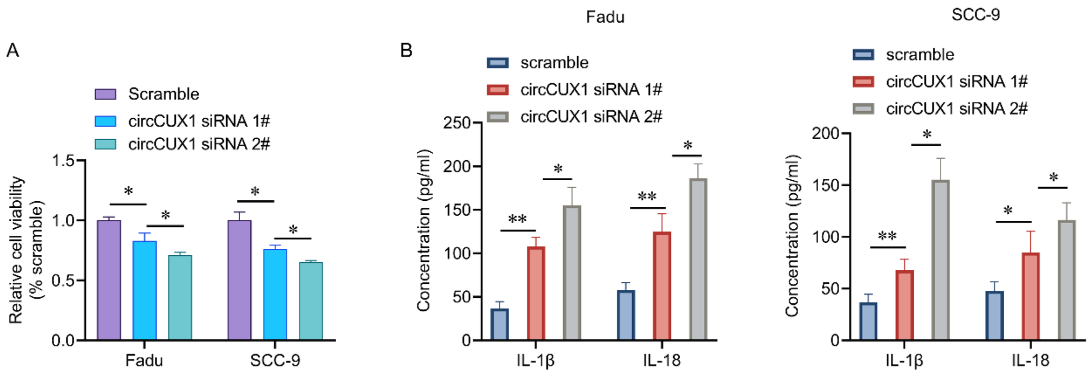

Supplementary Figure 4 The effects of circCUX1 siRNA transfection on CUX1 mRNA expression. qRT–PCR analysis for the expression of CUX1 mRNA after circCUX1 siRNA transfection in Fadu and SCC-9 cells.
